# A bioavailable strontium isoscape for Western Europe: A machine learning approach

**DOI:** 10.1371/journal.pone.0197386

**Published:** 2018-05-30

**Authors:** Clement P. Bataille, Isabella C. C. von Holstein, Jason E. Laffoon, Malte Willmes, Xiao-Ming Liu, Gareth R. Davies

**Affiliations:** 1 Department of Earth and Environmental Sciences, University of Ottawa, Ottawa, Canada; 2 Department of Geological Sciences, University of North Carolina, Chapel Hill, N.C., United States of America; 3 Department of Earth Sciences, Faculty of Science, Vrije Universiteit Amsterdam, The Netherlands; 4 Faculty of Archaeology, Leiden University, Leiden, The Netherlands; 5 Department of Wildlife, Fish, and Conservation Biology, University of California Davis, Davis, C.A., United States of America; California State University Northridge, UNITED STATES

## Abstract

Strontium isotope ratios (^87^Sr/^86^Sr) are gaining considerable interest as a geolocation tool and are now widely applied in archaeology, ecology, and forensic research. However, their application for provenance requires the development of baseline models predicting surficial ^87^Sr/^86^Sr variations (“isoscapes”). A variety of empirically-based and process-based models have been proposed to build terrestrial ^87^Sr/^86^Sr isoscapes but, in their current forms, those models are not mature enough to be integrated with continuous-probability surface models used in geographic assignment. In this study, we aim to overcome those limitations and to predict ^87^Sr/^86^Sr variations across Western Europe by combining process-based models and a series of remote-sensing geospatial products into a regression framework. We find that random forest regression significantly outperforms other commonly used regression and interpolation methods, and efficiently predicts the multi-scale patterning of ^87^Sr/^86^Sr variations by accounting for geological, geomorphological and atmospheric controls. Random forest regression also provides an easily interpretable and flexible framework to integrate different types of environmental auxiliary variables required to model the multi-scale patterning of ^87^Sr/^86^Sr variability. The method is transferable to different scales and resolutions and can be applied to the large collection of geospatial data available at local and global levels. The isoscape generated in this study provides the most accurate ^87^Sr/^86^Sr predictions in bioavailable strontium for Western Europe (R^2^ = 0.58 and RMSE = 0.0023) to date, as well as a conservative estimate of spatial uncertainty by applying quantile regression forest. We anticipate that the method presented in this study combined with the growing numbers of bioavailable ^87^Sr/^86^Sr data and satellite geospatial products will extend the applicability of the ^87^Sr/^86^Sr geo-profiling tool in provenance applications.

## Introduction

Strontium isotope ratios (^87^Sr/^86^Sr) are a commonly used geochemical tracer to investigate provenance of modern and ancient organisms and materials in archaeology, ecology, and forensics [1,2]. ^87^Sr/^86^Sr varies predictably between different geologic provinces, based on their age and composition, and through weathering is transferred into the hydrosphere and ecosphere. Animals take up ^87^Sr/^86^Sr from the environment and diet and incorporate this ratio into their skeletal and organic tissues. Many organic tissues of animals also grow continuously (e.g., otoliths, hair, teeth, bones, tusks, feathers) and record time-series of ^87^Sr/^86^Sr variations that can be used to reconstruct movement or migration patterns of individuals or entire populations. For instance, the ^87^Sr/^86^Sr ratio has successfully been used in many archeological studies to distinguish between locals and non-locals population, in forensics to track the origin of illicit products or human remains, in food science to certify food origin or in ecology to track modern and ancient animal migration [3–5]. The growing interest in applying ^87^Sr/^86^Sr ratio as a geolocation tool resides primarily in its unique pattern of geographic variations [6]. Geolocation information derived from traditional isotope systems will always be limited because of their broad gradients on the Earth’s surface (e.g., hydrogen and oxygen isotope systems) [1] and the difficulty in accurately predicting their high resolution spatiotemporal variations (e.g., carbon and nitrogen isotope systems). The ^87^Sr/^86^Sr ratio displays a high resolution but predictable scalar spatial pattern following geological regimes and limited temporal variability. As such, spatiotemporal patterns of ^87^Sr/^86^Sr values in the geosphere, ecosphere and hydrosphere provide precise and unique geolocation potential for provenance studies.

The basis of ^87^Sr/^86^Sr geolocation is to compare the ^87^Sr/^86^Sr profile of a sample of interest with that of a ^87^Sr/^86^Sr baseline to estimate geographic origin. This geographic assignment can be performed using two different approaches: the nominal approach and the Bayesian continuous approach [7]. In the nominal approach, the study area is divided into small blocks of potential areas of origin defined through a priori knowledge. The ^87^Sr/^86^Sr values in these small blocks are calculated as the mean value and variance of a series of local bioavailable ^87^Sr/^86^Sr data analyzed within each block. The ^87^Sr/^86^Sr value of the sample of interest is then compared to these possible a priori locations using a classification tree to generate the geographic assignments. In the Bayesian continuous approach, ^87^Sr/^86^Sr values of the sample of interest are compared to models predicting the ^87^Sr/^86^Sr values over the entire study area. These continuous predictive models also include spatial explicit uncertainty assessment allowing the evaluation of the probability of origin of a sample at each location relative to all other locations. Since Sr isotopes are distributed continuously throughout the environment, and since there is often overlap in ^87^Sr/^86^Sr ratios among geographically distinct locations (further complicating a priori group determination), the continuous probability approach makes geographic assignments more realistic and preferable. However, applying this Bayesian continuous approach of geographic assignment requires the development of accurate predictive ^87^Sr/^86^Sr models with spatially explicit uncertainty assessments [7]. These type of models have been developed for riverine environments and have been used successfully within a continuous probability framework, for example to manage a population of salmon across an entire watershed [8,9]. In terrestrial environments, however, the application of this powerful continuous assignment approach is hampered by the low accuracy of predictive ^87^Sr/^86^Sr models and their inability to provide spatially explicit uncertainty assessment [10]. Consequently, improving the accuracy of the predictive ^87^Sr/^86^Sr models for terrestrial ecosystems is an essential step to unlock the potential of the ^87^Sr/^86^Sr geolocation tool in terrestrial provenance studies.

Several approaches have been tested in recent years to develop regional-scale predictive ^87^Sr/^86^Sr maps for terrestrial environments. These models are either created by spatially interpolating empirical ^87^Sr/^86^Sr data [11–14] or by developing process-based spatial models predicting ^87^Sr/^86^Sr ratios on a given substrate [6,15–17]. Empirical interpolation methods rely on analyzing a large number of ^87^Sr/^86^Sr ratios to estimate the “bioavailable Sr pool” (the Sr pool locally available to ecosystems). This bioavailable Sr pool integrates Sr from different local sources and its resulting ^87^Sr/^86^Sr value is assumed to estimate the ^87^Sr/^86^Sr signature of local ecosystems. The choice of a reference substrate to represent this bioavailable Sr pool is non-trivial, as different sample materials can incorporate and integrate Sr at different spatial and temporal scales and from different sources [4,18–21]. The most common substrates used to represent the local bioavailable Sr pool include ^87^Sr/^86^Sr analyses of surface water, which integrates Sr sources at the watershed scale [22], soil leachates, which reflect local soil mineralogy [23], flora, which can uptake Sr from different soil depths depending on the rooting zone [12,13,23–26], fauna with limited feeding ranges, which source Sr from their local water and food [13,24,27] or combinations of these sample types. These bioavailable data can then be interpolated often in combination with other auxiliary variables (e.g. geological maps, sea salt aerosol deposition, landcover) to further refine the empirical ^87^Sr/^86^Sr prediction [11,14,25,28]. However, the accuracy of these empirical interpolation methods is generally low due to the non-normal distribution of ^87^Sr/^86^Sr data and the non-continuous scalar patterning of ^87^Sr/^86^Sr variability. As a result, these empirical models still require very high sampling density to produce spatial ^87^Sr/^86^Sr predictions at the regional scale [14], limiting their application. Process-based spatial ^87^Sr/^86^Sr models rely on our current knowledge of Sr isotopes cycling from rocks to hydro- and ecosystems. The starting point of these predictive efforts is to model ^87^Sr/^86^Sr variations in bedrock using the age and/or lithology from geological maps [16]. The propagation and mixing of this geologically-derived Sr into the hydrosphere and ecosphere is further predicted through modeling of chemical weathering, atmospheric deposition and soil mixing processes [15–17]. The models are validated using bioavailable ^87^Sr/^86^Sr datasets. However, the predictive power of these process-based models has remained limited due to the low resolution of regional geological maps in many countries and the complexity of integrating the multiple factors (e.g. geomorphology, pedology, hydrology, climate) and sources (geological, atmospheric, biological) influencing the ^87^Sr/^86^Sr variations at different spatial scales [17].

In this work, we combine process-based model products and regression techniques to model ^87^Sr/^86^Sr variations across Western Europe and overcome, by statistical means, the present lack of quantitative understanding of the controls of the bioavailable ^87^Sr/^86^Sr variability at regional scales. While we test several regression techniques, we focus primarily on random forest algorithms. Random forest [29] is an ensemble, multiple decision tree classifier that has been demonstrated to be highly accurate, adaptable and interpretable to map environmental variations and processes. The reasoning behind using random forest regression for mapping ^87^Sr/^86^Sr variations is that the algorithm can integrate non-normal, binary, continuous, categorical and non-independent predictor variables into a unified framework and is relatively insensitive to outliers. The objectives of this work are to generate more accurate bioavailable ^87^Sr/^86^Sr prediction and uncertainty maps for Western Europe to facilitate the application of the Bayesian continuous assignment approach in this key region of the world. Generating such a model will allow the application of the ^87^Sr/^86^Sr geolocation across western Europe for a variety of high-profile forensic, ecological and archeological applications from certifying wine origin to tracing the movement of ancient Neanderthal populations.

## Materials and methods

### Study area and target variables

**Fig 1:**
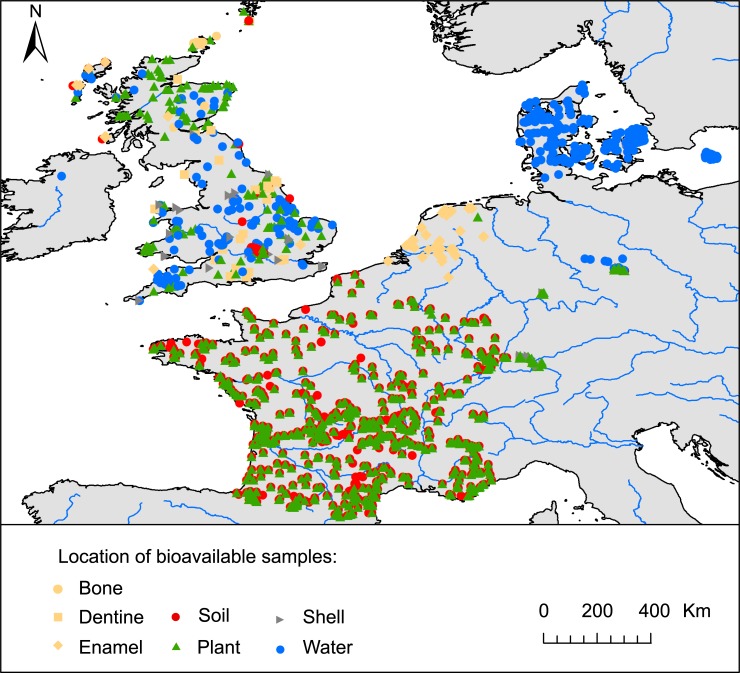
Sample locations and types of data included in the compilation dataset. Coastlines and rivers are from http://www.naturalearthdata.com/.

Georeferenced bioavailable ^87^Sr/^86^Sr data are only available for a few countries in Europe. The dataset used in this study is a compilation of 2551 published ^87^Sr/^86^Sr analyses from 1400 georeferenced locations in France, Great Britain, the Netherlands, Germany and Denmark (Fig 1) [12,21–23,28,30–35]. Data from plants, small animals (bone, dentine, enamel, snail shell), soils, rocks and surface waters were included as they estimate the local bioavailable Sr; aerosol dust (n = 2) and insect samples (n = 1) were excluded as the Sr from these substrates originate from non-local Sr sources (i.e., distant arid regions for dust, non-local feeding areas for flying insects). There are further bioavailable ^87^Sr/^86^Sr datasets in existence, for example from archaeological [36], hydrological [37] or food authenticity [11] studies, but these were not used because they either did not provide geographic coordinates or the raw ^87^Sr/^86^Sr data and/or targeted a sample type that was not considered sufficiently location-specific (e.g., large migratory mammal integrates Sr from different sources along their migratory pathways). Geographic coordinate systems differed between studies; all were converted to decimal latitude and longitude. Additional bioavailable ^87^Sr/^86^Sr data from agricultural and grazing soils has been generated by the GEMAS project [38] though not yet fully published, and is not included in the model.

### Auxiliary variables

#### Predictive models

We used the published model by Bataille et al. (2014) [15] and applied it to the study area. Briefly, the bedrock model is based on the radiogenic equation that specifies that the ^87^Sr/^86^Sr ratio in a given rock progressively increases due to the decay of rubidium -87 into strontium-87 through time. At present day, the ^87^Sr/^86^Sr ratio in a rock is a function of both its rubidium to strontium ratio (Rb/Sr), which controls the rate of radiogenic decay and its age. The bedrock model uses lithological and age information from geological maps to parameterize the age and Rb/Sr ratios of individual rock units and apply the radiogenic equation. The bedrock model predicts the median, quartile 1 and quartile 3 ^87^Sr/^86^Sr values of each rock unit of a geological map at present-day. We applied this model in western Europe using the GLiM geological map as primary source of geological information. For more information on the exact parameterization steps of the bedrock model, refer to the supplementary material in [15] including Table A1 and A3.

#### Other auxiliary data

Covariates were selected to represent factors of bioavailable ^87^Sr/^86^Sr variations according to Capo et al. (1998) [39]: parent rock, aerosols, biological processes, relief, climate, and water dynamics. Bioavailable ^87^Sr/^86^Sr variations are also controlled by processes that occur over timescales ranging from years (erosion, aerosol inputs) to millions of years (geology, topography). Consequently, we only used biological, hydrological and climatic datasets that represent multi-year averaging and with global coverage so that our method can be applied in other regions (Tables 1 and 2).

**Table 1:** List of bedrock model outputs from [15] used in the regression. D = discrete.

Variables	Description	Resolution	Type	Source
**r.m1**	Median bedrock model	1 km	D	This study
**r.srsrq1**	Quartile 1 bedrock model	1 km	D	This study
**r.srsrq3**	Quartile 3 bedrock model	1 km	D	This study

**Table 2:** List of geological, climatic, environmental and topographic variables used in the regression. D = Discrete; C = Continuous; GLiM = Global Lithological Map; CCSM.3 = Community Climate System Model 3; SRTM = Shuttle Radar Topography Mission.

Variables	Description	Transformation	Resolution	Type	Source
**r.xx** **r.litho**	GLiM 1^st^ lithological class attribute GLiM 2^nd^ lithological class attribute		1 km	D	[40]
**r.maxage_geol.t**	GLiM age attribute	Log	1 km	D	[40]
**r.minage_geol.t**	GLiM age attribute	Log	1 km	D	[40]
**r.meanage_geol.t**	GLiM age attribute	Log	1 km	D	[40]
**r.age**	Terrane age attribute		1 km	D	[41]
**r.salt.t**	CCSM.3 simulation	Log	1.4°×1.4°	C	[42]
**r.dust.t**	Multi-models average	Log	1°×1°	C	[42]
**r.elevation**	SRTM		90 m	C	[43]
**r.cec**	Cation Exchange Capacity		250 m	C	[44]
**r.ph**	Soil pH in H_2_O solution		250 m	C	[44]
**r.phkcl**	Soil pH in KCl solution		250 m	C	[44]
**r.clay**	Clay (weight %)		250 m	C	[44]
**r.silt**	Silt (weight %)		250 m	C	[44]
**r.sand**	Sand (weight %)		250 m	C	[44]
**r.orc**	Soil organic carbon (weight %)		250 m	C	[44]
**r.bulk**	Bulk density (kg m^−3^)		250 m	C	[44]
**r.bouguer**	WGM2012_Bouguer		2 min	C	[45]
**r.soilthickness**	Global soil thickness		1 km	C	[46]
**r.map.t**	Mean annual precipitation (mm.yr-^1^)	Log	30-arc sec	C	[47]
**r.pet**	*Global Potential Evapo-Transpiration *		30-arc sec	C	[48]
**r.ai**	*Global Aridity Index*		30-arc sec	C	[48]
**r.lc**	Global Land Cover 2009		300 m	D	[49]

### Modelling

#### Random forest regression

**Fig 2:**
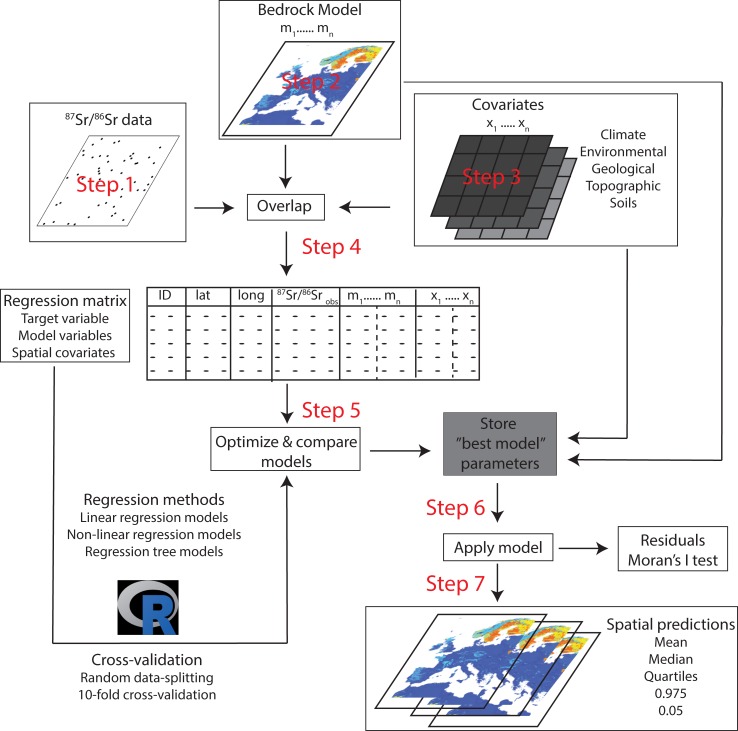
Flowchart summarizing the workflow used for the regression analysis. Tested covariates are given in Tables 1 and 2. The R script is provided in the supporting information.

In this study, our primary goal was to test the potential of random forest regression algorithms to overcome some of the difficulties in predicting ^87^Sr/^86^Sr variations on the Earth’s surface. While we use other algorithms as a point of comparison (Fig 2; Table 3), for conciseness, we only describe the random forest algorithm in detail (for information about the other algorithms refer to the associated R packages given in Table 3). Random forest belongs to the family of tree-based machine learning algorithms. It predicts a response from a set of predictors by creating multiple decision trees and aggregating their results [29]. The decision trees themselves are constructed through recursive partitioning of a bootstrapped subset (bagging) of the training data (root node). The root node is split by defining an optimal threshold based on a randomly selected subset of predictor auxiliary variables to provide two resulting data partitions, each with the greatest purity (the least variation in the target variable). This process is then repeated successively on each data partitions until the terminal nodes are reached. The terminal node is reached, and the mean value of the target variable recorded, when the number of samples present in the last partition reach the value specified by the user.

**Table 3:** Statistical algorithms tested in this study.

Name	Model	R package
**Linear regression models**		
**Glm**	Generalized linear model	Base
**svmLinear**	Support Vector Machines with Linear Kernel	Kernlab
**Gam**	Generalized Additive Model using Splines	Mgcv
**Non-linear regression models**		
**Nnet**	Neural Network	Nnet
**svmRadial**	Support Vector Machines with Radial Basis Function Kernel	Kernlab
**Regression tree models**		
**Gbm**	Stochastic Gradient Boosting	Gbm
**rf (and qrf)**	Random forest (and quantile random forest)	Randomforest (and ExtraTrees)
**Geostatistical models**		
**Ok**	Ordinary Kriging	Autokrige

#### Workflow for regression modeling

Spatial prediction, fitting of models and generation of maps, was implemented in R (the script is available in the supporting information) following the workflow of the caret package [50]. The process of generating the ^87^Sr/^86^Sr isoscape consisted of the following steps (Fig 2):

- Project all auxiliary data into WGS84-Eckert IV and resample at 1km resolution (bilinear interpolation for continuous variables and nearest neighbor interpolation for categorical variables).
- Extract covariate values at the geolocation of samples and build the regression matrix.
- Train the spatial prediction models using the caret package.
- Apply spatial prediction models using raster brick (covariates).
- Assess accuracy using cross-validation.

We compared random forest regression with other regression methods available in the *caret* package [50] and with ordinary and universal kriging implemented in the *automap* package [50]. A series of algorithms were tested (Table 3) that belong to different categories including:

- Linear regression models that aim to explain the spatial distribution of a dependent variable by means of a linear combination of predictors.
- Non-linear regression models that are similar to linear regression models but do not require linear relationship to be known *a priori*.
- Regression trees that split the training dataset into sub-groups having similar response values.
- Ordinary kriging that predict the spatial variations of a dependent variable by modeling spatial autocorrelation.

Models were computed for the total dataset, and for sub-datasets for each sample category (plants, soils, waters).

#### Model optimization and predictors selection

We fitted each of the above models (Table 3) for a combination of tuning parameters using the train function of the caret package [50]. The train function selected the optimal parameters in the different regression using root mean squared error (RMSE) as primary metric and a 10-fold repeated cross-validation scheme with 5 repetitions using 80% of the data for training at each iteration. The average RMSE values obtained from the k-fold cross-validation is used as the primary metric to compare the predictive power of models with each other. A potential issue, however, is that the predictive power of linear and non-linear regression models is often decreased by the multicollinearity of predictors and by the non-normal distribution of predictors and target variables. Regression trees are not as sensitive to these issues and can generally produce better models without pre-processing of the input variables. While this is convenient, we wanted to assess the full potential of linear and non-linear models to predict ^87^Sr/^86^Sr variations. To limit the problem of multicollinearity for linear models, generalized least squares (GLS) was conducted to identify significant predictors needed to model ^87^Sr/^86^Sr variations using generalized linear models following Beguería et al. (2013) [51]. To verify that the non-normality of predictor and target variables did not significantly decrease the performance of the linear and non-linear models, we tested different transformations of the covariates when fitting linear models. For random forest, we optimized our model using the variable selection procedure of the *VSURF* package [52], focusing on variable selection for prediction. Variable importance and predictive relationships with the target variable were further visualized using the variable importance purity measure and partial dependence plots to visualize the importance of different predictors. Variable selection was not independently optimized for other non-linear models, only the variables selected by VSURF as optimum for random forest were submitted to the other models. As a last step, spatial autocorrelation in relation to geographic distances between sites was examined for the residuals of all created models using Moran I statistics.

#### Isoscape

The ^87^Sr/^86^Sr isoscape maps were produced using the best performing regression random forest model constructed on the whole dataset (n = 1400 individual sites) and the associated predictors. While random forest provides a mean ^87^Sr/^86^Sr prediction using the selected predictors, there are no built-in features to assess spatially explicit model uncertainty other than the cross-validated RMSE. Spatial uncertainty assessment is critical for using isoscapes in continuous-probability surface models of geographic assignment [10]. To circumvent this issue, we used quantile random forest regression to generate spatially explicit raster of uncertainty around the prediction. Where random forest takes the mean of the outputs of the ensemble of decision trees as the final prediction, quantile regression forests also take specified quantiles from the outputs of the ensemble of decision trees. The mean of the outputs of the ensemble of decision trees was used as the predicted value, and for each prediction the 2.5th and 97.5th percentiles of the ensemble were used as the lower and upper limits of a 95% prediction interval. We generated ^87^Sr/^86^Sr predictions (mean and median) and uncertainty (width of the 95% prediction interval) at 1 km^2^ resolution.

## Results and discussion

### Limitations of the bioavailable dataset

The bioavailable dataset compiled in this study is highly variable in terms of sample density, geographic distribution and sample type (Fig 1). The compiled dataset is largely a reflection of varying national and scientific interest and funding for archaeological provenance research. Sample collection strategies and types (waters, rocks, soils, dust, plant tissue and animal tissue) differ greatly between studies, reflecting research study context, e.g., systematic/opportunistic sample collection, the scale of the archaeological enquiry, local and regional geological context, and current land use.

Variability in sample type occurs across the study area, e.g., there are no soil samples analyzed from mainland Denmark and no shell samples from France. This is problematic because the different sample types characterize different parts of the total Sr available to the biosphere, deriving from a combination of mineral, atmospheric and anthropogenic sources [4]. Subsoil and topsoil can have different ^87^Sr/^86^Sr ratios due to differential mineral weathering, deposition types and soil management [53]. In addition, plants with different rooting depths draw on differing Sr pools [18,54]. Animals differ in the nature, spatial scale and temporal scales of their Sr inputs: e.g., snail shells reflect soil carbonate values [21] or rainwater values [12] rather than bulk local soil values. Different bodies of surface water also differ in the nature and scales of their Sr inputs [55]. Soil ^87^Sr/^86^Sr ratios are dependent on the leaching methodology used [22], which differs between studies included in this dataset. The regional variability in sample types across the study area therefore means that regionally different fractions of local total bioavailable Sr contributions are reflected in the dataset. This is not ideal, as it will bias the predictions derived from this dataset towards the dominant sample type used in each region. The forthcoming GEMAS dataset [38] may provide more systematic coverage and a homogeneous dataset of soil ^87^Sr/^86^Sr ratios should substantially improve the quality of bioavailable isoscapes for Western Europe. Sampling in the GEMAS project is, however, also biased as it is restricted to agricultural and grazing soils, which are likely to have received exogenous addition of Sr in the form of fertilizer, lime or animal waste [21,22].

**Fig 3:**
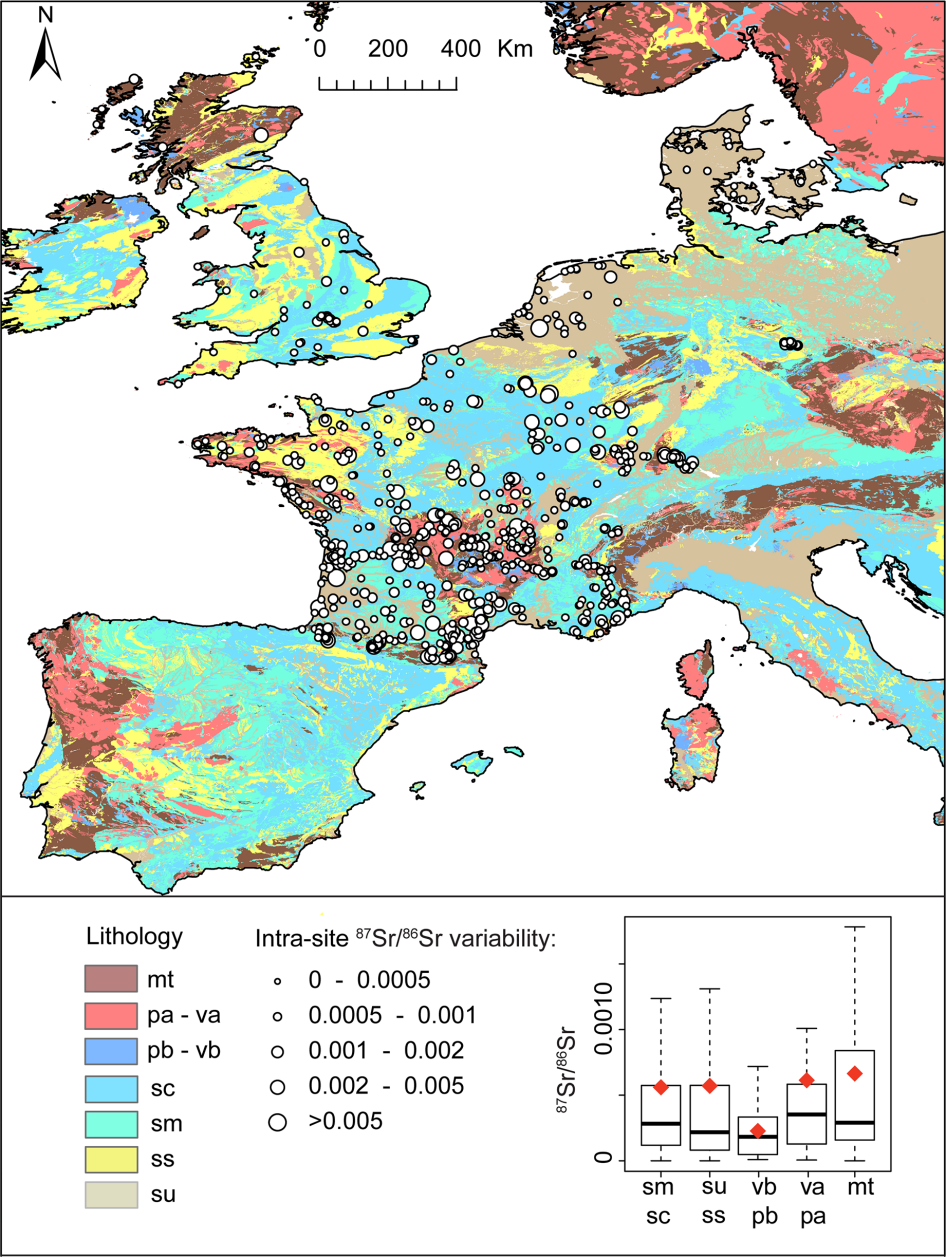
Within-population ^87^Sr/^86^Sr uncertainty visualization. Bubble map of the standard deviation of ^87^Sr/^86^Sr analyses of different substrates from the same site plotted over the lithological map of Western Europe from the GLiM database. A boxplot in the bottom-right corner shows the variability of ^87^Sr/^86^Sr ratios by geological group with the thick black line representing the median value, the box representing the quartiles and the tails representing minimum and maximum value. We added the mean value as a red diamond for comparison. mt = metamorphic rocks; pa = felsic plutonic rocks; va = felsic volcanic rocks; pb = mafic plutonic rocks; vb = mafic volcanic rocks; sm = mixed sediments; sc = carbonate sediments; ss = siliciclastic sediments; su = unconsolidated sediments. Coastline features are from http://www.naturalearthdata.com/.

In the present study, we estimate the ^87^Sr/^86^Sr variability between sampled substrates (within-population uncertainty) by calculating the median standard deviation of ^87^Sr/^86^Sr ratios between samples at each location where more than one substrate was collected (median = 0.0003). This uncertainty is primarily controlled by the local bedrock ^87^Sr/^86^Sr ratios (Fig 3). The difference between ^87^Sr/^86^Sr ratios from different substrates at the same site tends to be higher in complex geological regions such as felsic and metamorphic units (Fig 3) or when the ^87^Sr/^86^Sr ratio of the local bedrock differ significantly from the ^87^Sr/^86^Sr ratios of other local sources of Sr (e.g., aerosols or local erosion). While in the long term developing substrate specific models would probably be more appropriate, the uncertainty represented by using multiple substrates is less than 1% of the total range of bioavailable ^87^Sr/^86^Sr ratios of the dataset. In this study, we opted to use the whole bioavailable dataset with multiple substrates and we used the median of the ^87^Sr/^86^Sr ratios when several samples were collected from the same site. Considering the small variance of ^87^Sr/^86^Sr values between substrates, using multiple substrates will not significantly increase the uncertainty but will allow testing the random forest model on a larger dataset with a broader spatial extent. As such, using multiple substrate appears appropriate for the resolution and accuracy targeted by this study but the approach should be re-evaluated for more local and targeted studies.

### Model performance

#### Comparison of random forest regression with other regression methods

**Fig 4:**
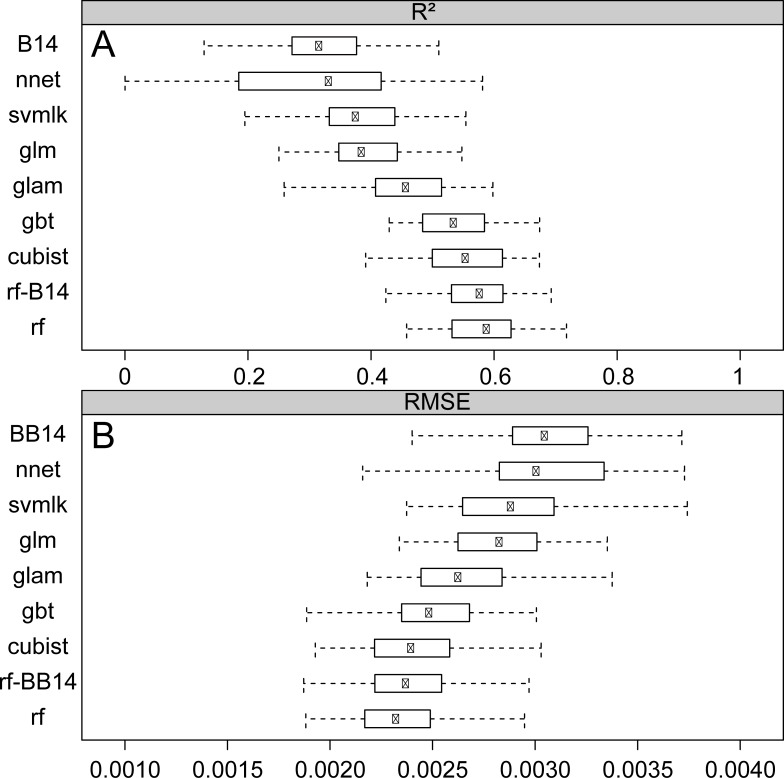
Model performance metrics. Summary of tested regression algorithm performance after 10-fold cross validation with 5 repetitions using: A. boxplots of the correlation coefficient (R^2^) and B. boxplots of the root mean square error (RMSE). The regression methods are sorted by their predictive power from top to bottom. For model abbreviations please refer to Table 3. rf-B14: Random forest regression without Bataille et al. 2014 products; B14: Random forest regression using only Bataille et al. (2014)’s model products (Table 1).

Comparison of cross-validated RMSE and correlation coefficient (R^2^) values between random forest, and other regression algorithms indicates that random forest regression is the best performing regression algorithm to predict ^87^Sr/^86^Sr variations over Western Europe (rf model on Fig 4). The model performance of the 10-fold cross-validated model (R^2^ = 0.58 and RMSE = 0.0023) significantly outperforms all other tested regression algorithms (Fig 4). Fig 4 shows that, algorithms from the machine learning class perform significantly (Wilcox test p-value << 0.05), better than linear, and non-linear regressions at predicting ^87^Sr/^86^Sr variations over Western Europe. Among the machine learning algorithms, although random forest shows the best predictive power and significantly outperforms other methods (Wilcox test p-value<0.05), generalized regression boosting tree and cubist regressions also perform well at predicting ^87^Sr/^86^Sr variations (Fig 4B). The better performance of machine learning algorithms probably stems from their ability to make use of different types of predictor variables (categorical and continuous) and from their insensitivity to non-normal distribution and outliers of the training dataset. Both advantages are critical when trying to predict ^87^Sr/^86^Sr variations that are usually positively skewed and display multiscale patterning of variations [16].

The above comparisons used models built with variables selected by the VSURF process for rf models, which might not be optimal for linear models. We independently optimized the generalized linear regression models, by log-transforming the target variable and the covariates and by recursive elimination of multicollinear covariates. The best generalized linear regression model gave a RMSE of 0.0028. Counterintuitively, transformation of auxiliary and target variables decreased model fit (RMSE = 0.004).

#### Comparison of random forest regression with ordinary kriging

We used the self-optimizing algorithms from the R *automap* package to interpolate ^87^Sr/^86^Sr variations using an ordinary kriging model. With a RMSE of 0.0026, the ordinary kriging prediction accuracy is significantly lower than that of all the machine learning algorithms but outperforms parametric regression models. The better performance of machine learning algorithms over ordinary kriging is not surprising as kriging methods assume second order stationarity and spatial autocorrelation. Those assumptions are generally incorrect for ^87^Sr/^86^Sr variation that primarily depends on the discrete bedrock unit distribution. Some additional work could be attempted to circumvent this issue. For instance, applying a kriging based on geological units or groups of units might help train better variograms and make better use of categorical geological variables [14].

#### Comparison of random forest regression with Bataille et al. 2014’s model

Random forest significantly outperforms the Bataille et al. (2014) [15]process-based model (RMSE = 0.0031) for predicting ^87^Sr/^86^Sr variations over Western Europe (BB14 model on Fig 4). This result is unsurprising as Bataille et al. (2014)’s model is used as an auxiliary variable in the random forest regression (Fig 2 and Tables 1 and 2). We tested a random forest regression model that did not include Bataille et al. (2014)’s model products (rf-B14 model on Fig 4). This model gives slightly lower performance (RMSE = 0.0024) than the regression model trained using Bataille et al. (2014)’s model products (RMSE = 0.0023). However, the small difference in performance suggests that random forest regression is a powerful algorithm to integrate complex, non-linear relationship using geological auxiliary variables (i.e., age and lithology layers from the GLiM database) to model geologically-derived ^87^Sr/^86^Sr variability.

#### Comparison of random forest regression by sample category

Random forest regression was independently performed on the plant, soil and waters subsets of the bioavailable dataset (n = 800, 689 and 395 locations respectively) to compare with the results of the complete dataset (n = 1400 locations, i.e., some locations are sampled for multiple substrates). The VSURF-optimized random forest models including Bataille et al. (2014)’s products remained the best-performing regression technique for all substrates. However, in comparison with the whole dataset, the performance was worse for the plant (0.0026) and soil (0.0028) subsets and better for the waters subset (0.0016). The improvement in predictive power between the water model and those for plants and soils is probably partly related to the differences in Sr contributions between sample types: surface water integrates Sr inputs over larger spatial scales than plants and soils decreasing the isotopic variability [4]. The difference in predictive power is also dependent on the biased spatial distribution of different sample substrates (Fig 1) and their relationship to geological complexity [4]. For instance, a large proportion of the water samples come from Denmark, which has relatively homogenous geology, compared to France from where the bulk of the plant and soil samples derive from varied geological units (Fig 1).

### Random forest regression model

In the following section, we further explore the predictive power, variable importance and uncertainty of the best performing random forest regression using the whole bioavailable dataset (rf model on Fig 4).

#### Performance of the random forest regression

**Fig 5:**
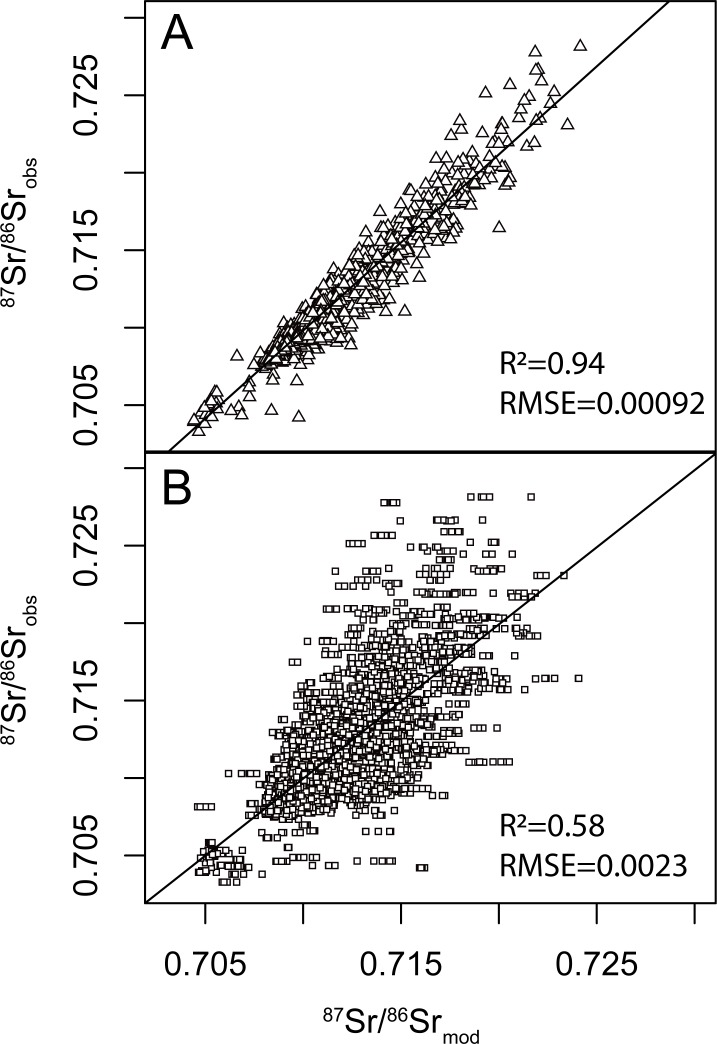
Scatter plots of observed vs. predicted ^87^Sr/^86^Sr ratios. A. Training dataset, B. 10-fold cross-validation testing dataset. R^2^ = coefficient of determination; RMSE = root mean square error.

The trained random forest regression model resulting from the k-fold cross-validated procedure explains 94% of the variance with a RMSE of 0.0009 over the training datasets (Fig 5A) and 58% of the variance with a RMSE of 0.0023 over the testing datasets (Fig 5B). The remaining training error is higher than the within-population uncertainty (see section 3.1) but represents less than 3% of the full range of observed bioavailable ^87^Sr/^86^Sr ratios of the dataset. The low training error demonstrates that random forest regression can fit highly accurate models for bioavailable ^87^Sr/^86^Sr variations. The actual model uncertainty is calculated using the k-fold cross-validated RMSE over the testing datasets (Fig 5B). The value of 0.0023 represents less than 8% of the full range of observed bioavailable ^87^Sr/^86^Sr ratios over the study area. This is a significant improvement over other regression methods and models (Fig 4). Increased data coverage should further increase the accuracy and resolution of ^87^Sr/^86^Sr predictions. Keeping the k-fold cross-validated RMSE over the testing datasets below 10% of the observed ^87^Sr/^86^Sr range should constitute a reasonable goal to integrate ^87^Sr/^86^Sr isoscapes within the continuous-probability surface model approach [7,56].

#### Variable importance assessment

**Fig 6:**
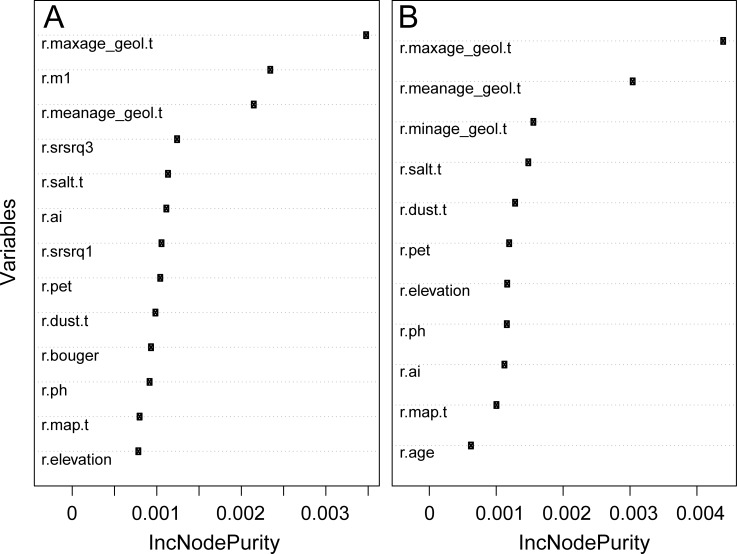
Variable importance plot for the best performing random forest models. A. Variable importance plot for the best performing random forest model (rf model on Fig 3) after selection of variables using the VSURF algorithm; B. Variable importance plot for the random forest regression model without Bataille et al. (2014)’s model products (rf-B14 on Fig 3) after selection of variables using the VSURF algorithm. Refer to Tables 1 and 2 for description of variables.

The regression forest model for ^87^Sr/^86^Sr finds Bataille et al. (2014)’s data products and geological variables from the GLiM database to be the most important predictors of ^87^Sr/^86^Sr variations (Fig 6). When Bataille et al. (2014)’s data products are not used in the regression, age of geological units (minimum, maximum and mean age layer in Table 2) are the most important predictors selected by the VSURF algorithm (Fig 6B). When Bataille et al. (2014)’s data products are included as covariates, the maximum and mean age of geological units from the GLiM database remain among the top predictors selected by the VSURF algorithm (Fig 6A). This observation suggests that Bataille et al. (2014)’s model does not make full use of the information contained in the GLiM database and that random forest regression is able to incorporate additional information from those layers. Overall, this exercise confirms the predominance of geological variables in controlling ^87^Sr/^86^Sr variations on the surface [17]. Other important predictors of ^87^Sr/^86^Sr variations include dust and seasalt aerosol deposition (r.dust.t and r.salt.t), elevation (r.elevation and r.bouguer), climate variables (r.pet, r.map and r.ai) and soil pH (r.ph) (Fig 6A and 6B).

In plant, soil and water sample subsets, VSURF optimization of variable selection also consistently selected GLiM age attributes and the Bataille et al. (2014)’s model products as the most important predictive variables. When these products were excluded from model optimization, GLiM age attributes remained the most important variables for all subcategories. Other variables differed in importance between sample categories. The plant and water sample subsets are very similar to the whole dataset in terms of variable selection and importance. The importance of elevation in the whole dataset is probably related to soil data, as this is the only subset in which this auxiliary variable is selected by the VSURF optimization procedure. Potential evapo-transpiration (r.pet) appeared important only in the plant and water subsets. Those observations suggest differences in the control of Sr cycling between substrates. The relationship between potential evapo-transpiration and ^87^Sr/^86^Sr in plants and water might be linked to the important role of water table height in controlling the mixing of Sr sources [18]. The ^87^Sr/^86^Sr of the most surficial reservoirs, such as water and plants, will be particularly sensitive to small changes in potential evapo-transpiration as they would affect groundwater circulation and control the contribution of bedrock Sr vs. aerosol Sr to the surface [39].

The independent exploration of variable importance using the generalized linear model regression also confirmed the significance of Bataille et al. (2014)’s data products and geological variables (transformed and untransformed) from the GLiM database. Other variables of importance included climate variables (r.map and r.pet) and soil pH (r.phk1 and r.ph). For models built without Bataille et al. (2014) data products, RMSE was always lower than for the model which included them, regardless of variable transformation.

#### Relationship between covariates and ^87^Sr/^86^Sr variations

**Fig 7:**
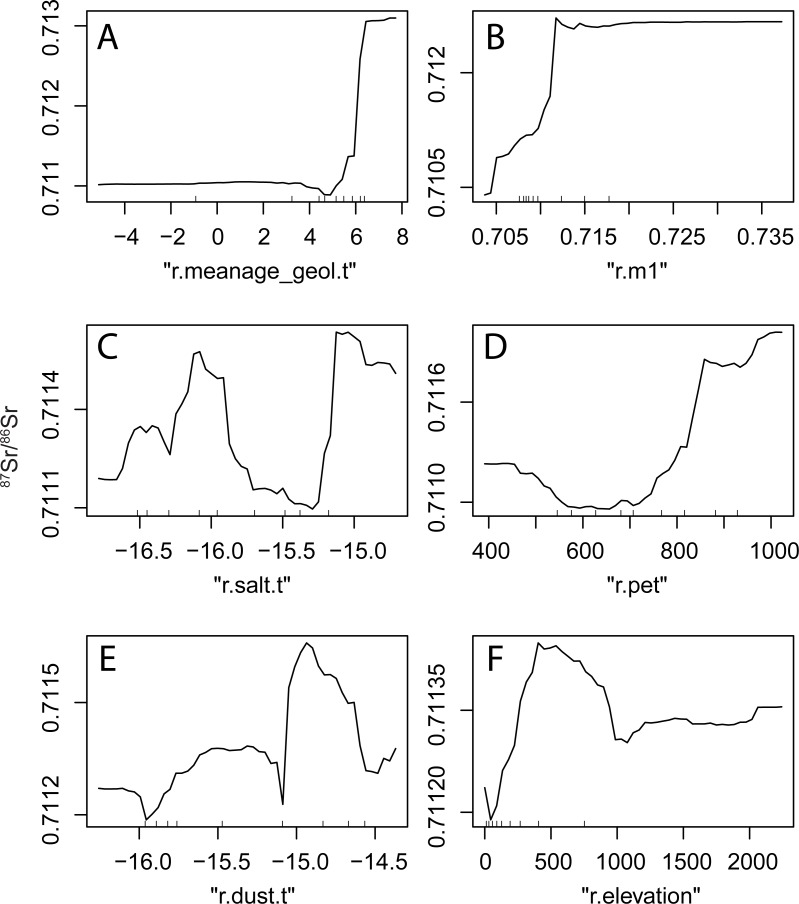
Partial dependence plots for some important variables. A. Log-transformed maximum age of geological units (R.meanage_geol.t) from GLiM database (r.meanage_geol.t in Table 2), B. Median prediction of Bataille et al. (2014)’s model applied to Western Europe (r.m1 in Table 1), C. Log-transformed values of Sr flux from sea salt aerosol deposition in t.km^-2^.a^-1^ (r.salt.t in Table 2), D. Potential Evapo-transpiration (r.pet in Table 2), E. Log-transformed values of Sr flux from dust aerosol deposition (r.dust.t in Table 2) and F. Elevation (r.elevation in Table 2). Refer to Tables 1 and 2 for description and sources of each covariate. Hash marks along the x axis show covariate sample decile values.

We used a partial dependence plot to further investigate the form of the relation between bioavailable ^87^Sr/^86^Sr ratios and covariates (Fig 7). The visualization of the partial dependence plots demonstrates the advantages of using random forest to predict ^87^Sr/^86^Sr variations rather than linear regression models. In linear or non-linear regression, relationships between variables are reduced to parametric relationships, and cannot account for non-continuous, discrete or non-monotonous relationships between variables. Conversely, the random forest regression applied in this work shows multiple forms of linear, non-linear and threshold-based relationships (Fig 7).

^87^Sr/^86^Sr variations are exponentially correlated with the log-transformed maximum age of geological units (r.meanage_geol.t) (Fig 7A). However, the age of geological units only becomes an important control of ^87^Sr/^86^Sr variations for older rock units (age >> 100 Ma) (Fig 7A). This observation is in line with the 48.8 billion year half-life of ^87^Rb such that ^87^Sr/^86^Sr ratios of rocks are significantly affected over time periods of several tens of millions of years (Fig 7A). The relationship between ^87^Sr/^86^Sr variations and the mean ^87^Sr/^86^Sr prediction from Bataille et al. (2014)’s model (r.m1) is linear between 0.704 and 0.713 but remains constant above this value (Fig 7B). This observation suggests that this version of Bataille et al. (2014)’s model cannot be efficiently used to inform prediction of high ^87^Sr/^86^Sr ratios. This is expected as in Bataille et al. (2014), an interpolation step is used to model the spatial variability of ^87^Sr/^86^Sr ratios by lithology. In this work, we bypass this interpolation step and use an averaging parameterization more comparable to Bataille and Bowen (2012). Bataille and Bowen noted that when using an averaging approach for the parameterization, the lack of spatiotemporal resolution of geological maps, the rapid intra-unit geochemical variations of felsic rock units, and the skewed distribution of ^87^Sr/^86^Sr ratios in those rock types lead to large underestimates in the ^87^Sr/^86^Sr predictions of high ^87^Sr/^86^Sr ratios.

The relationship between ^87^Sr/^86^Sr variations and the log-transformed sea salt (r.salt.t) and dust (r.dust.t) deposition is more complex (Fig 7C and 7E). In general, ^87^Sr/^86^Sr ratios decrease with increasing sea salt deposition (Fig 7C), probably reflecting the low ^87^Sr/^86^Sr ratios of marine-derived Sr. Conversely, ^87^Sr/^86^Sr ratios increase with increasing dust deposition (Fig 7E), probably reflecting the contribution of radiogenic Sahara dust to ecosystems in Europe [57]. However, for both these variables, the form of the relationship with ^87^Sr/^86^Sr ratios is complex, reflecting the geological, pedological and climatological processes controlling the incorporation and contribution of aerosol derived Sr to ecosystems [39].

Climatic and topographic variables also contribute to ^87^Sr/^86^Sr variability as suggested by the importance of the potential evapo-transpiration (r.pet) and elevation variables (r.elevation) (Fig 7D and 7F). ^87^Sr/^86^Sr ratios increase with increasing potential evapo-transpiration (Fig 7D), possibly reflecting the increased contribution of radiogenic aerosol dust in regions with higher potential evapo-transpiration [58]. ^87^Sr/^86^Sr data have a complex relationship with elevation with highest ^87^Sr/^86^Sr ratios for intermediate elevation (500 m to 1,000 m). This relationship is probably due to a geographic bias in the compiled dataset. Most of the samples located within the 500 m to 1,000 m range of altitude are from the eroded remnant of the Variscan and Caledonian orogenies (i.e., Massif Central, Brittany, Western England and Scotland). These old radiogenic mountain ranges are oversampled in comparison with the younger mountain ranges (e.g., Pyrennees, Alps).

#### Understanding the remaining spatial uncertainty

In addition to the cross-validated RMSE, our approach uses quantile regression forest to provide a conservative measure of the range of ^87^Sr/^86^Sr ratios at each pixel. The 95% uncertainty interval (2.5^th^ to 97.5^th^ forest quantiles) is unique at each pixel and is controlled by the strength of the dependency to auxiliary variables. The cross- validation showed that this 95% uncertainty interval is an accurate probabilistic estimate for ^87^Sr/^86^Sr ranges as more than 90% of the testing data fell within the interval. As such, the quantile regression maps can be used to generate envelopes around the prediction and to apply continuous-probability surface model approaches [10].

**Fig 8:**
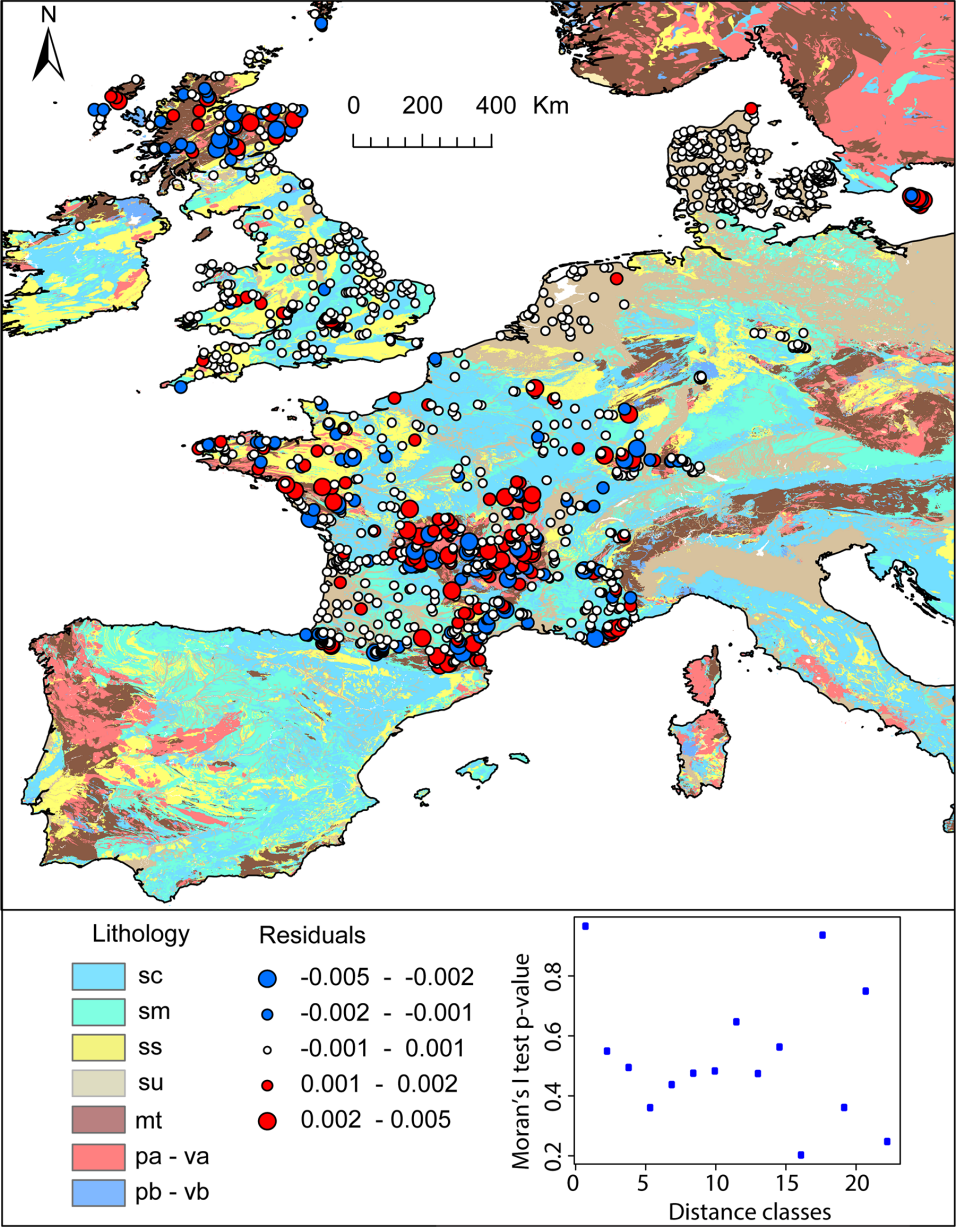
Bubble map of ^87^Sr/^86^Sr residuals for the training dataset plotted over the lithological map of Western Europe from the GLiM database. In the bottom-right corner, a plot shows the p-value of the Moran’s I test for different distance class with p-value >0.05 indicating no spatial autocorrelation. mt = metamorphic rocks; pa = felsic plutonic rocks; va = felsic volcanic rocks; pb = mafic plutonic rocks; vb = mafic volcanic rocks; sm = mixed sediments; sc = carbonate sediments; ss = siliciclastic sediments; su = unconsolidated sediments. Coastline features are from http://www.naturalearthdata.com/.

The spatial correlograms do not show any spatial correlation in the residuals of the random forest regression (Moran’s test I p-value>>0.05 for all distance classes) suggesting that random forest regression has successfully modeled the spatial dependence of ^87^Sr/^86^Sr variations (Fig 8). The residual ^87^Sr/^86^Sr variations can be attributed to processes that are spatially random at the scale of the study area and at the resolution studied. However, a closer look at the performance of the random forest regression reveals that, while no spatial correlation can be deciphered in the residuals, the RMSE values vary for different geological classes (Fig 8). Most of the extreme residual values are located within metamorphic and felsic rock units (Fig 8). The average ^87^Sr/^86^Sr ratios of felsic units and of their minerals is on average higher but more variable than other rock types because minerals contained in felsic rocks have a wide range of Rb/Sr ratios [16]. As felsic and metamorphic rocks are more resistant to weathering than other rock types, they also tend to have a higher average age on the Earth’s surface as observed for western Europe [59]. When soils develop on one of these rock units, the ^87^Sr/^86^Sr values of the bioavailable Sr coming from the bedrock will change through time reflecting the progressive evolution of the soil mineralogical composition with differential weathering [60]. For instance, at equal age, the ^87^Sr/^86^Sr ratios of highly weathered granite will be higher than in other granite because most plagioclases with lower ^87^Sr/^86^Sr values have been weathered while biotite with higher ^87^Sr/^86^Sr values is more resistant [61]. Differential weathering processes or soil age can lead the ^87^Sr/^86^Sr values of a soil to differ largely from its bulk ^87^Sr/^86^Sr composition. These processes are not accounted for in our modeling approach and limit the accuracy of prediction, particularly for felsic and metamorphic rock units. Similarly, the RMSE for siliciclastic sediment rock units is higher than for other sediment types. Siliciclastic sediments are composed of minerals from different provenance and with different age. Depending on their geological history, the ^87^Sr/^86^Sr range of these individual grains can be large and can differ significantly from the depositional age of the sediment (i.e., recycling). Additionally, the different minerals composing these siliciclastic rock units can weather at different rates, further complicating the prediction of ^87^Sr/^86^Sr values. A primary focus to improve ^87^Sr/^86^Sr isoscapes is to find appropriate auxiliary variables to model the mineralogy of rock units and/or the provenance of sediment minerals. However, global geospatial datasets providing some information about bedrock mineralogy are not currently available and limit our ability to represent the geological ^87^Sr/^86^Sr variability at high resolution. To improve the model prediction for ^87^Sr/^86^Sr values in siliciclastic sediment, it might be beneficial to use an approach that is capable of learning higher-order context (learning textures and spatial patterns rather than just point proprieties) to account for fluvial transport and sediment provenance. However, this type of model would also increase the effective degrees of freedom within each model, and would require more training data. Consequently, the most reasonable approach to improve the learning of the distribution of bioavailable ^87^Sr/^86^Sr on rock units displaying a large range and extreme ^87^Sr/^86^Sr ratios is to sample these rock units at higher density.

Another important source of remaining uncertainty for this regression model are the inaccuracies of geological maps. Geological maps are generated by multiple geologists, agencies and countries that use different mapping schemes, and nomenclatures. They contain some obvious inconsistencies, as evidenced by the clear discrepancies of geological units at some country boundaries. Geological map products strongly contribute to the ^87^Sr/^86^Sr prediction in the random forest regression. The use of such geological map products adds a layer of uncertainty that even the best machine learning algorithms will have difficulty to resolve. While efforts to harmonize geological maps are underway (onegeology project), replacing geological maps with satellite or aerial covariate data would greatly enhance the consistencies of ^87^Sr/^86^Sr predictions. For instance, the replacement of geological maps by aerial gamma ray survey products as auxiliary variable could resolve some of the inconsistencies of lithological observations. However, to date, geological auxiliary variables are not available at global scale and can only serve as covariates when training more local ^87^Sr/^86^Sr isoscapes.

To assess spatially the influence of aerosol deposition on our model predictions, we compared the residuals of our optimized random forest model with two other random forest models for which sea salt deposition and dust deposition were not included as auxiliary variables. We found that the sea salt deposition strongly contributes to the ^87^Sr/^86^Sr prediction in areas dominated by igneous or metamorphic rock units including Scotland, Britany, Cornwall, the Jura, the Pyrenees and the Massif central. The strong influence of sea salt aerosols in these igneous and metamorphic regions was expected as these geological units have low Sr content, low chemical weathering rate and their ^87^Sr/^86^Sr values diverge strongly from seawater ^87^Sr/^86^Sr ratio. Under these conditions, sea salt aerosols can contribute significantly to base cations in soils, particularly when mean annual precipitation is high [57]. Dust deposition strongly contributes to the ^87^Sr/^86^Sr prediction in the south of France and Scotland. The strong influence of dust deposition in southern France likely reflects the high level of deposition from local and Sahara dust sources in this region, which can contribute up to 30% of the soil base cations [57]. The strong influence of dust deposition to ^87^Sr/^86^Sr prediction in Scotland is probably an artifact of the model and reflects the overall lower contribution of Sahara dust in Scotland relative to other regions in southern Europe [42].

#### Comparing random forest ^87^Sr/^86^Sr isoscapes with other models

**Fig 9:**
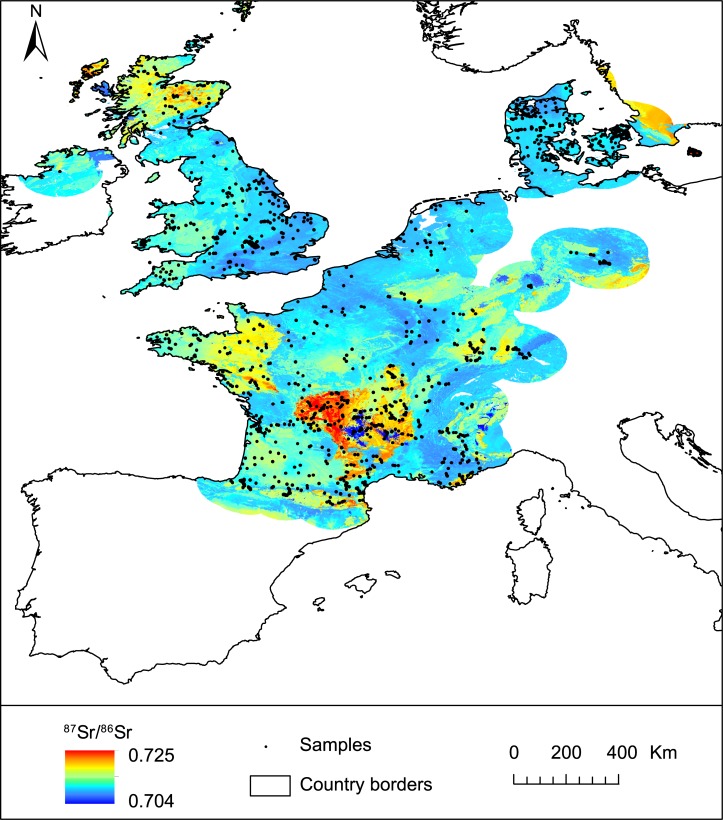
Random forest regression bioavailable ^87^Sr/^86^Sr map for Western Europe. Coastline features are from http://www.naturalearthdata.com/.

The ^87^Sr/^86^Sr isoscape produced using random forest regression has a spatial resolution governed by that of the auxiliary variables (Fig 9). In this study, we used a resolution of 1 km^2^ for all covariates to limit computing time. The maps generated resolve the spatial distribution of ^87^Sr/^86^Sr variations in much more detail than traditional empirically-based ^87^Sr/^86^Sr models, which are limited by the spatial density of the bioavailable ^87^Sr/^86^Sr sampling [11,12,22]. At large scales, the ^87^Sr/^86^Sr variations from the random forest model follow discrete variations reflecting the age and lithology of geological units. Superimposed on the geological patterns, the ^87^Sr/^86^Sr isoscape predicts a gradient of decreasing ^87^Sr/^86^Sr ratios from the coast to more inland locations in Brittany, Wales and Scotland. This pattern has been previously recognized as demonstrating the influence of sea salt aerosols on bioavailable ^87^Sr/^86^Sr ratios [12].

**Fig 10:**
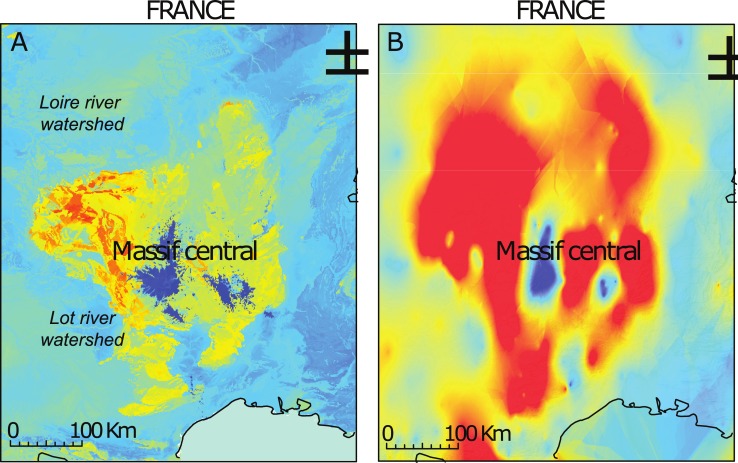
Bioavailable ^87^Sr/^86^Sr maps for the Massif Central. A. Random forest regression bioavailable ^87^Sr/^86^Sr map; B. Ordinary kriging bioavailable ^87^Sr/^86^Sr map. Color scale is the same as Fig 9. Coastline features are from http://www.naturalearthdata.com/.

Zooming in on a particular region such as the Massif Central (Fig 10), the pattern of ^87^Sr/^86^Sr variations can be interpreted in more detail to verify that the modeled ^87^Sr/^86^Sr variations fit with our current knowledge of Sr cycling on the surface [39]. The increase in prediction detail is evident when comparing the ^87^Sr/^86^Sr isoscape produced by random forest regression (Fig 10A) and those produced by ordinary kriging (Fig 10B). At the finer scale, we observe the presence of directional linear features within geological units that follow geomorphological features such as river valleys. The influence of fluvial sediment transport on ^87^Sr/^86^Sr variations has been previously recognized [62], in particular, river valleys often accumulate sediments from different upstream sources that have distinct ^87^Sr/^86^Sr ratios from the local rock units. This is the case for the Loire and Lot rivers that show higher ^87^Sr/^86^Sr ratios than the local marine sediments of the Aquitaine and Parisian basins respectively [55]. Both rivers carry a proportion of sediment from the radiogenic rocks of the Massif Central, which are deposited downstream in basins dominated by unradiogenic marine sediments.

In summary, the ^87^Sr/^86^Sr isoscape derived from random forest regression generates the theoretically-expected multi-scale ^87^Sr/^86^Sr patterning, with discrete patterns following distinct geological units, directional features following geomorphological processes (e.g., river valleys) and continuous gradients following climate variables (e.g., aerosol deposition). This multi-scale patterning is in sharp contrast with the relatively continuous ^87^Sr/^86^Sr variations produced by ordinary kriging that can only map ^87^Sr/^86^Sr variations as broad gradients with prediction rapidly deteriorating away from the bioavailable sampling sites. The predicted surface presented here is a distinct step forward for the generation of maps of ^87^Sr/^86^Sr variation on the Earth’s surface, as it enables higher resolution mapping without requiring major sample collection campaigns.

#### Applicability of the ^87^Sr/^86^Sr isoscape

The Western European isoscape generated in this study provides, to date, the highest-resolution ^87^Sr/^86^Sr predictions and includes a spatially explicit assessment of uncertainty. Together these two products give the possibility to integrate terrestrial ^87^Sr/^86^Sr data from modern and ancient organic and inorganic materials collected across Western Europe into continuous-probability surface models for geographic assignment. Using this framework, ^87^Sr/^86^Sr data can be coupled with other isotope systems (e.g., H and O) to enhance the accuracy of provenance assignment in forensic, food science, ecology and archeology.

It should also be remembered that creating a single bioavailable Sr map for a given area is an artificial exercise because the “bioavailable” fraction varies between different parts of the biosphere [4]. Though it has higher resolution than the maps previously generated by other methods, this isoscape is still limited by the present availability and consistency of bioavailable ^87^Sr/^86^Sr data across Western Europe. As such, we encourage researchers to recalibrate their own isoscape using the framework presented here but by using empirical datasets, covariate resolution and spatial extent more appropriate to their particular research question. The current map is only valid for areas where data coverage was sufficient to calibrate and test the prediction (France, Great Britain, Netherlands and Denmark). The accuracy and resolution of the strontium isoscape could be further improved by integrating new data and we strongly urge researchers to publish all environmental ^87^Sr/^86^Sr ratios with full latitude and longitude data. The development of online repositories to store bioavailable data and potentially model ^87^Sr/^86^Sr variations [23] could be a great step-forward in this direction.

Provenance studies have underlying assumptions specific to the sample type and the question being addressed. The map presented here is best suited as a broad scale approach to exclude provenance areas and inform where targeted sampling for a specific research question should occur. When the samples in question exhibit a limited range over which they incorporate bioavailable strontium, as is the case for plants, soils, and animals with small feeding ranges, the map can further be used to predict areas of origin. For provenance studies that investigate large scale animal movement and human migrations, it should be remembered that large mammals incorporate and average strontium across a larger range. In these cases, it does not make sense to match the sample to a specific geologic unit but this map can still be used to identify large scale migratory patterns. Further modeling is required to predict ^87^Sr/^86^Sr in rivers and the map will not perform well for those types of provenance studies. Finally, the strontium isotope data can be easily combined with auxiliary information, including field data and other isotope systems, and can thus provide a valuable geochemical tracer in a larger context.

## Conclusions

In this study, we assessed the performance of random forest regression to generate a bioavailable ^87^Sr/^86^Sr isoscape for Western Europe. We highly encourage the application of random forest regression to generate ^87^Sr/^86^Sr isoscapes at local to global scales because:

1. Random forest regression, in conjunction with process-based models and geospatial environmental auxiliary data, outperformed other mapping methods in predicting ^87^Sr/^86^Sr variations.
2. Random forest regression provides a flexible framework to integrate different types of auxiliary variables which are required to model the multi-scale patterning of ^87^Sr/^86^Sr variability.
3. Random forest regression yielded measures of variable importance and visualization tools that make the ^87^Sr/^86^Sr isoscape easier to interpret.
4. Quantile regression forest can generate conservative uncertainty assessment, which is a critical component for integrating ^87^Sr/^86^Sr variations in continuous-probability surface models for geographic assignment.

Improving the accuracy, resolution and spatial extent of Sr isoscape using random forest regression will require the compilation and generation of additional bioavailable ^87^Sr/^86^Sr data coupled with further integration of environmental covariates from new geospatial products.

## Supporting information

S1 File R script. (R)
